# Deep learning [^18^F]-FDG-PET/CT‑based algorithm for tumor burden estimation in metastatic melanoma patients under immunotherapy

**DOI:** 10.1016/j.ctro.2025.101063

**Published:** 2025-10-27

**Authors:** Lorenzo Lo Faro, Hubert S. Gabryś, Simon Burgermeister, Daniel Abler, Maksym Fritsak, Maiwand Ahmadsei, Ciro Franzese, Adrien Depeursinge, Michel A. Cuendet, Stephanie Tanadini-Lang, Panagiotis Balermpas, Marta Scorsetti, Matthias Guckenberger, Sebastian M. Christ

**Affiliations:** a Dept. of Radiation Oncology, University Hospital and University of Zurich, Zurich, Switzerland; b Dept. of Radiotherapy & Radiosurgery, IRCCS Humanitas Hospital, Via Manzoni, Rozzano, Milan, Italy; c Dept. of Oncology, Geneva University Hospitals and University of Geneva, Geneva, Switzerland; d Institute of Information Systems, University of Applied Sciences Western Switzerland, Sierre, Switzerland; e Dept. of Biomedical Sciences, Humanitas University, Via Rita Levi Montalcini, Pieve Emanuele, Milan, Italy; f Department of Nuclear Medicine and Molecular Imaging, Lausanne University Hospital, Lausanne, Switzerland; g Swiss Institute of Bioinformatics, Lausanne, Switzerland; h Dept. of Physiology and Biophysics, Weill Cornell Medicine, NY, USA

**Keywords:** Deep-learning, Autosegmentation, Metastatic melanoma

## Abstract

**Background and purpose:**

Artificial intelligence is increasingly used in radiation oncology, yet its application for tumor burden (TB) estimation remains limited. This study evaluated the performance of a [^18^F]-fluorodeoxyglucose positron emission tomography/computerized tomography ([^18^F]-FDG-PET/CT)-based deep learning model, PET-Assisted Reporting System (“PARS”, *Siemens Healthineers*), for lesion detection, segmentation, and TB estimation in patients with metastatic melanoma undergoing immunotherapy.

**Materials and methods:**

This retrospective study included 165 stage IV melanoma patients who underwent [^18^F]-FDG-PET/CT imaging prior to immunotherapy. Gross tumor volumes were segmented using PARS and compared with manual delineations performed by radiation oncologists. Performance was assessed through lesion detection metrics (precision and recall), individual lesion volume agreement, and overall TB estimation accuracy.

**Results:**

PARS demonstrated an overall recall (sensitivity) of 68.9 %, though with modest precision (46.8 %). Performance was location-dependent, with highest precision observed for lung lesions (74.0 %) and lowest for bone lesions (32.9 %). For lesions detected by both methods, PARS tended to underestimate lesion volumes by an average (median) of 0.9 cc (median relative percentage difference (MRPD) =  −34.3 %), with a good agreement (intraclass correlations coefficient (ICC) = 0.77). The global TB in the whole cohort was overestimated by 28.3 %, but patient-level TB was on average (median) underestimated by 1.1 cc (MRPD =   −18.4 %) with high variability with a median absolute relative percentage difference (MARPD) = 68.6 %) and poor agreement (intraclass correlation coefficient (ICC) = 0.28).

**Conclusions:**

PARS shows potential for treatment decision support with moderate accuracy in lesion detection and lesion volume estimation, but demonstrates significant variability in TB estimation, highlighting the need for further model refinements before clinical adoption.

## Highlights

• This study evaluates a deep learning model (PARS) for lesion detection and tumor burden estimation in metastatic melanoma.
• PARS detected 68.9% of expert-identified lesions but with a modest precision of 46.8%.
• Lung lesions showed highest precision (74.0%), while bone lesions had the lowest (32.9%).
• Lesion volume estimates had good agreement (ICC = 0.77) but were generally underestimated.
• Tumor burden estimation was highly variable and poorly correlated with expert assessments (ICC = 0.28).

## Introduction

In recent years, artificial intelligence (AI) has emerged as a transformative force in radiation oncology, revolutionizing key areas such as the identification and contouring of organs at risk (OARs) and tumors [[1], [2], [3], [4], [5], [6], [7], [8], [9]], the development of radiotherapy treatment plans [[10], [11], [12]], and the assessment and prediction of treatment responses [[13], [14], [15], [16]]. Deep learning (DL) models, a subset of AI tools, have demonstrated the ability to streamline these processes, offering significant time savings and enhancing workflow efficiency across clinical settings.

Manual tumor segmentation, a critical step in radiotherapy planning and treatment monitoring, remains a time-consuming, labor-intensive task, that requires the expertise of highly trained clinicians. Studies evaluating automated segmentation have reported time savings ranging from 27 % in prostate cancer to as much as 93 % in head and neck cancers [17,18]. Despite these advances, tumor segmentation presents unique challenges compared to OAR segmentation. These challenges arise from tumor-specific factors, including size variability, e.g., in brain tumors, the intricate nature of surrounding anatomical structures, e.g., in head and neck tumors, and the frequent lack of clear tumor boundaries, e.g., in abdominal cancers.

The complexity of tumor segmentation further increases in oligo- and polymetastatic disease, where lesions appear in diverse anatomical locations and tumor environments and can be challenging to differentiate from neighboring regions of physiologically high glucose intake. In such settings, an effective automated segmentation tool must, once again, overcome significant heterogeneity in lesion size, shape, and surrounding tissues. A reliable and efficient model capable of accurately identifying individual lesion size changes, detecting new metastatic lesions, and quantifying total tumor burden (TB) could provide substantial benefits in radiation oncology throughout longitudinal patient monitoring, enabling rapid treatment adaptations, and ultimately improving patient outcomes [19].

Metastatic melanoma, the deadliest form of skin cancer, despite recent therapeutic advances, represents an ideal candidate for exploring AI-driven tumor segmentation and TB estimation. Mortality from melanoma is predominantly due to metastatic spread, which is typically assessed during follow-up via serial computer tomography (CT) or [^18^F]-fluorodeoxyglucose positron emission tomography [^18^F]-FDG-PET/CT imaging. Although automated tumor segmentation approaches have been studied in other malignancies, research in metastatic melanoma remains limited. Recently, *Dirks et al. (2022)* introduced a PET-based DL software for metastatic melanoma lesion detection and contouring, achieving promising results in segmentation accuracy and minimizing false positive and false negative rates [20].

This study aims to evaluate the performance of a novel PET-based DL software [[21], [22], [23], [24]], PET-Assisted Reporting System (“PARS”, *Siemens Healthineers,* [25,26]), for the detection and delineation of metastatic melanoma lesions. Additionally, it investigates the tool’s ability to provide precise TB estimations both at baseline and during follow-up imaging. To our knowledge, this study represents the largest cohort to date focusing on the objective quantification of TB in metastatic melanoma, contributing valuable insights to the field of AI applications in radiation oncology.

## Materials and methods

### Study design

Retrospective, single-center diagnostic accuracy study. The automated PET/CT segmentation model (PARS) was compared with expert manual segmentation as the reference standard. Primary outcome was lesion-level detection. The secondary outcome was agreement for lesion size and patient-level burden.

### Patient cohort

This retrospective study included 165 patients diagnosed with stage IV melanoma who underwent treatment with either single (anti-PD-1; *Nivolumab*, or, anti-CTLA4; *Ipilimumab*) or dual checkpoint inhibition (anti-PD-1/anti-CTLA-4; *Nivolumab*/*Ipilimumab*) or a combination with an indoleamine 2-3-diosygenase (IDO) inhibitor (IDO1i; E*pacadostat*) between 2013 and 2019 at the Comprehensive Cancer Center Zurich (CCCZ). The study adhered to Good Clinical Practice (GCP) guidelines and the Declaration of Helsinki and was approved by the local ethics committee, the Kantonale Ethikkommission Zürich (KEK), approval number #2019-01012. Written informed consent was obtained from all participants.

Patient inclusion criteria were a confirmed metastatic melanoma diagnosis, presence of at least one non-brain lesion at the baseline, and availability of [^18^F]- FDG-PET/CT imaging. Patients were excluded if they lacked baseline or follow-up imaging, had only contrast-enhanced CT imaging, presented with brain metastases as the sole metastatic site, or had baseline lesions smaller than 0.5 cc. A detailed summary of patient demographics and clinical characteristics is provided in Table 1.

**Table 1:** Patients’ characteristics.

**All patients**	165
**Age (years)**	
Median	67
Q1–Q3	53–74
Range	28–93

**Sex**	
Male	112 (67.9 %)
Female	53 (32.1 %)

**Type of ICI**	
aPD1	120 (72.7 %)
aCTLA4 + aPD1	43 (26.1 %)
aCTLA4	1 (0.6 %)
aPD1 + ID01i	1 (0.6 %)

**Number of lesions**	
Total	909
Median	3
Q1-Q3	1– 7
Range	1– 46

**Metastatic sites**	
Lymph node	300 (33.0 %)
Lung	168 (18.5 %)
Liver	116 (12.8 %)
Bone	110 (12.1 %)
Other	215 (23.7 %)

### Imaging protocols

All included patients underwent [^18^F]- FDG-PET/CT prior to treatment initiation. Imaging was conducted using standardized protocols across a single institution. [^18^F]-FDG doses were adjusted based on body weight or body mass index (BMI) (2.0–3.5 MBq per kg). The mean uptake time was 60 min.

The scans were acquired using a range of PET/CT scanners (GE Discovery MI (38 %), GE Discovery 690 (36 %), GE Discovery RX (12 %), GE Discovery STE (8 %), and other (6 %)). Pet images were reconstructed using OSEM variants with TOF and PSF when available in 63 % of studies. GE Q.Clear (BSREM) was used for the remaining 37 % of scans. In CT, no contrast was used. kVp was set to 120 kV and 140 kV in 78 % and 22 % of scans, respectively. Standard CT reconstruction kernel was used in 99 % of cases.

### Lesion segmentation

Ground-truth segmentation was performed manually in three dimensions by two experienced radiation oncologists following a common protocol and relying on radiological reports. CT and PET images were rigidly registered, the PET signal was used for localization, CT for tissue contouring. CT-based contours were propagated onto PET images. Spatial mismatches were corrected manually, and PET-based contours were refined to conform to PET signal intensities.

Automated lesion segmentation was performed using PARS. PET regions with standard uptake value (SUV) peak values exceeding the mean blood pool uptake (SUVBP) by more than two standard deviations (SDs) were identified and segmented using a 42 % threshold of the local SUV max. Lesions smaller than 0.5 cc were excluded from further analysis. The convolutional neural network within PARS classified segmented foci as “benign” (model output < 0.5) or “suspicious” (model output ≥ 0.5). In this study we focused on analyzing, PARS segmentations marked as “suspicious” and did not evaluate the “benign” ones.

### Statistical analysis

Manual segmentations served as the ground truth for evaluating the PARS model's performance. The PARS output included lesion segmentations and a probability score indicating the likelihood of each segmentation being “suspicious”. Each segmentation was classified as true positive or false positive based on comparison with expert annotations.

The accuracy of lesion detection was assessed using precision and recall (sensitivity). These performance metrics were computed for the entire cohort and further stratified by anatomical locations to assess region-specific performance variations. Additionally, precision and recall were evaluated across varying probability thresholds to illustrate the inherent trade-offs between sensitivity and specificity.

The accuracy of lesion detection count, individual lesion size estimation, and overall TB estimation by the PARS model were evaluated using several statistical measures: Median difference, median absolute difference (MAD), median relative percentage difference (MRPD), median absolute relative percentage difference (MARPD), and the intraclass correlation coefficient (ICC(3,1)) were calculated to quantify agreement and variability between PARS and expert measurements.

## Results

Experts identified a total of 955 lesions across 165 patients (Table 1). The PARS model identified 6456 benign and 1377 suspicious lesions, with the classification threshold set at the default value of 0.5 to distinguish between benign and suspicious lesions.

In several cases, multiple small segmentations by PARS corresponded to a single larger segmentation by the experts, or vice versa. To establish a consistent one-to-one correspondence between expert and PARS segmentations, smaller segmentations were merged. Following this process, the number of expert-defined lesions was reduced to 909, and the unique PARS segmentations were reduced to 6450 benign and 1337 suspicious lesions.

**Fig. 1:**
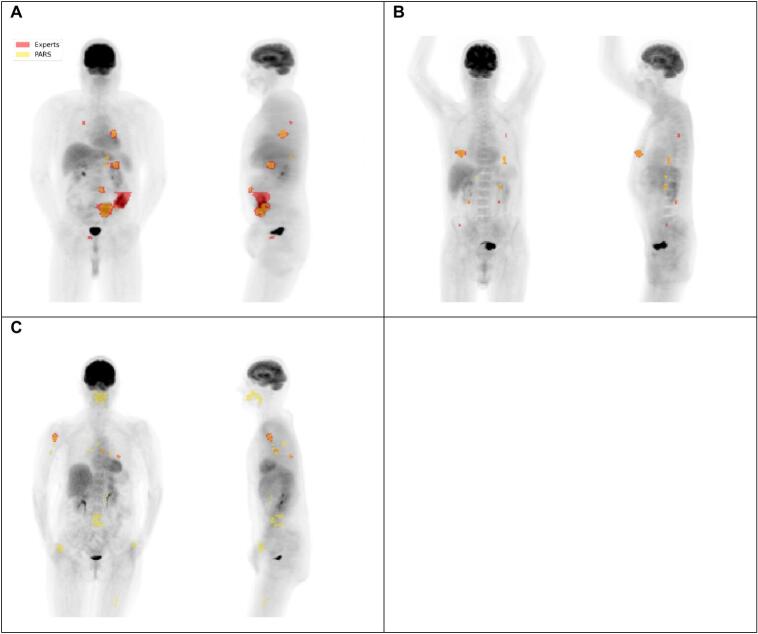
Examples of the differences between expert and PARS segmentations. A: Substantial underestimation of TB by PARS; B: Good match between experts and PARS; C: Substantial number of false positives and overestimation of TB.

Overall, PARS identified 1337 suspicious lesions, representing a 47.1 % overestimation compared to the experts' count. The model's overall precision was 46.8 %, indicating that more than half of the lesions detected by PARS were false positives (n = 711). The overall recall (sensitivity) was 68.9 %, as the model successfully detected 626 of the 909 lesions identified by experts (Table 2). Visual comparison of expert and PARS-based segmentations of a few selected cases is presented in Fig. 1.

**Table 2:** Precision and recall of the PARS model in detecting lesions overall and stratified by anatomical location. Precision represents the proportion of correctly identified lesions among all lesions identified by PARS, while recall indicates the proportion of true lesions identified by PARS. Bold values indicate the highest recall and precision among the evaluated anatomical sites.

**Location**	**Recall**	**Precision**
All	68.9 %	46.8 %
Lymph node	71.0 %	47.8 %
Bone	74.5 %	32.9 %
Liver	**81.9 %**	43.8 %
Lung	57.7 %	**74.0 %**

Lesion detection performance varied significantly depending on anatomical location (Table 2). The highest precision was observed for lung lesions (74.0 %), indicating effective identification with fewer false positives. In contrast, bone lesions had the lowest precision (32.9 %), reflecting a substantial number of false positives. Liver and lymph node lesions showed intermediate precision values of 43.8 % and 47.8 %, respectively. Despite its low precision, the model demonstrated relatively high recall for bone lesions (74.5 %), indicating good sensitivity, but difficulty in distinguishing true lesions from false positives.

**Fig. 2:**
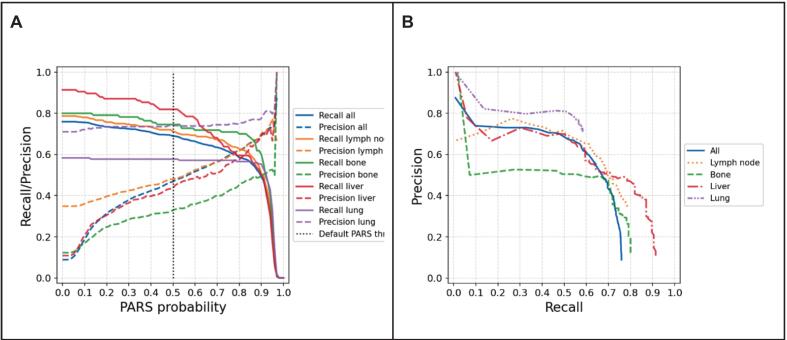
Precision and recall of PARS model. A: Precision and recall of the PARS model plotted against the PARS probability threshold for different tumor sites. B: Precision–recall curves for each site, illustrating the relationship between precision and recall across all possible probability thresholds.

Performance trade-offs between precision and recall at various lesion classification probability thresholds were also analyzed (Fig. 2). Increasing the threshold improved precision by reducing false positives, but simultaneously decreased recall, leading to fewer true lesions being identified. This trend highlights the inherent challenge in balancing sensitivity and specificity in automated lesion detection.

**Fig. 3:**
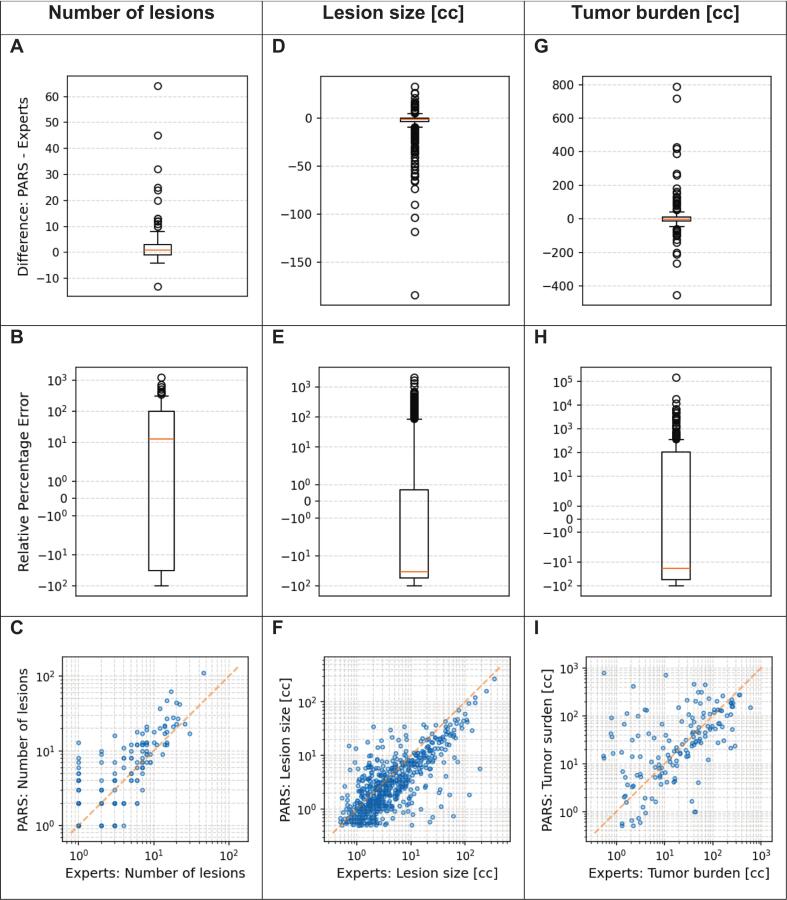
Comparison of PARS and expert assessments for three metrics: (A, B, C) the number of lesions, (D, E, F) the lesion size (in cc), and (G, H, I) the total tumor burden (in cc). The top row of boxplots (A, D, G) shows the absolute difference (PARS – Experts), while the middle row (B, E, H) shows the relative percentage error on a symlog scale. In each boxplot, the central line denotes the median, box edges indicate the interquartile range, and whiskers extend to 1.5 × IQR. Outliers are shown as open circles. The bottom row (C, F, I) displays scatter plots of PARS versus Experts, with the diagonal line indicating equality.

At the patient level, PARS overestimated the lesion count by an average of approximately one lesion per patient, albeit with substantial variability (Table 3, Fig. 3). The ICC for lesion counts per patient was 0.68, indicating moderate agreement between PARS and expert assessments.

**Table 3:** Agreement between the PARS model and expert annotations for lesion detection, individual lesion size estimation, and total tumor burden estimation. Metrics include median difference, median absolute difference (MAD), median relative percentage difference (MRPD), median absolute relative percentage difference (MARPD), and the intraclass correlation coefficient (ICC(3,1)) with 95% confidence intervals (CI).

	**Median difference**	**Median absolute difference (MAD)**	**Median relative percentage difference (MRPD)**	**Median absolute relative percentage difference (MARPD)**	**ICC (95 % CI)**
Lesion detection	1	2	12.5 %	57.1 %	0.68 (0.60– 0.76)
Lesion size [cc]	−0.9	1.5	−34.3 %	44.9 %	0.77 (0.55–0.88)
Tumor burden [cc]	−1.1	14.0	−18.4 %	68.6 %	0.28 (0.12–0.51)

For lesions detected by both PARS and experts (n = 626; 68.9 % of expert-identified lesions), PARS tended to underestimate lesion volume by an average of 0.9 cc (MRPD =  −34.3 %; Table 3). The ICC of 0.77 suggests relatively good agreement for individual lesion size estimates.

Stratified analysis by lesion location (Table S1), showed that the median absolute error was greatest for liver lesions (3.9 cc) and smallest for lung lesions (0.7 cc). Percentage-based analyses revealed low systematic bias, but high variability for liver lesions (MRPD =  −6.1 %, MARPD =  56.4 %), whereas lung lesions were consistently undersegmented with lower variability (MRPD =  −22.0 %, MARPD =  38.0 %). The highest ICC, indicating excellent agreement, was achieved for liver lesions (ICC =  0.90), whereas lung lesions had the lowest ICC =  0.52.

Across the entire cohort, PARS overestimated the total TB by 28.3 %. However, at the individual patient level, the model generally underestimated TB by an average of 1.1 cc (MRPD =  −18.4 %; Table 3, Fig. 3). The discrepancy between overall and individual-level findings results from high variability in estimates between patients. This variability was also reflected in a high median absolute difference (MAD) = 14.0 % and mean absolute relative percentage error (MARPE) = 68.6 %. The ICC for TB estimation was low (0.28), indicating poor overall agreement between PARS and expert assessments.

## Discussion

This study evaluated the performance of the PARS model for detecting, segmenting, and estimating TB in metastatic melanoma, focusing on its application in a real-world cohort. PARS achieved a recall of 68.9 %, identifying most true lesions, but its precision was limited (46.8 %) due to high FP rates. Anatomical site-specific performance varied, with lung lesions exhibiting the highest precision (74.0 %) and bone lesions the lowest (32.9 %), despite high recall (74.5 %) in the latter. Overall, the agreement between the PARS model and expert annotations demonstrated moderate to good performance in lesion detection (ICC =  0.68) and individual lesion size estimation (ICC =  0.77). However, the agreement for estimating total TB was considerably lower (ICC =  0.28), indicating limited reliability of the model in accurately assessing overall TB.

The high FP rate highlights a common challenge in DL-based segmentation, differentiating between pathological and physiological [^18^F]-FDG uptake [[4], [5], [6]]. Bone lesions, which exhibited particularly low precision, likely reflect the inherent complexity of bone imaging due to overlapping signals from bone marrow activity and metabolic processes. Similarly, lymph node segmentation may suffer from challenges in distinguishing benign hyperplasia from malignant involvement. However, it is important to bear in mind that only lymph node lesions > 0.7 cm were manually contoured by physicians. Both recall (68.9 %) and precision (46.8 %) in this study compare unfavorably to previous studies. For example, according to *Sibille et al. (2020)*, PARS achieved an area under the curve (AUC) of the receiver-operating characteristic of >0.95 for lesion classification and anatomic localization, and accuracies of 96 % for body part and 87 % for organ localization, respectively, in lymphoma and lung cancer cases, for which PARS was originally trained. Addressing some of these issues might involve refining the classification algorithm within PARS to incorporate multi-parametric features, such as lesion shape, heterogeneity, and proximity to critical anatomical landmarks, which could help improve specificity without sacrificing recall.

With respect to TB estimation, a similar observation can be made: *Capobianco et al.* (2021) found that TB estimation in lymphoma patients, for which the model was originally built, in this particular case with Diffuse Large B-Cell Lymphoma (DLBCL), was consistent with that obtained by experts and displayed significant prognostic value for both progression-free and overall survival [24]. In contrast, *Abler et al.* (2023) reported comparable results to ours in terms of detection precision (53 %) and negative-predictive-value (80 %) when assessing malignant melanoma lesions, even though the imaging data set was less granular (not by organ) and based on a review of a small set of automatic segmentations (24 images) [27]. Of note, the authors also found that detection accuracy improved when combining predictions at different time points. While PARS has not been trained extensively on metastatic melanoma patients, the variability in performance across anatomical sites nonetheless points to limitations in PARS' current approach to volumetric analysis. Smaller lesions and lesions with irregular boundaries, which were more prone to overestimation, may benefit from improved contour refinement algorithms. Incorporating edge-detection techniques and probabilistic models to delineate unclear boundaries could enhance segmentation accuracy [28]. Furthermore, using multi-modal imaging inputs—such as combining PET with MRI to provide more detailed soft-tissue contrast—could improve delineation in regions like the liver and lymph nodes [29].

Moreover, future iterations of PARS could leverage newer DL architectures such as transformer models, which have shown promise in other medical imaging applications [30]. Transformers are capable of modeling long-range dependencies, making them well-suited for detecting subtle differences in signal intensity and spatial relationships between lesions and surrounding tissues. Additionally, incorporating unsupervised or semi-supervised learning approaches could help overcome the scarcity of annotated data, particularly for rare or atypical lesion presentations [31].

Last, but not least, active learning, where the model queries clinicians to annotate uncertain cases during training, could also be implemented to iteratively improve PARS segmentation performance [32]. This approach would help the model learn from edge cases, such as atypical metastatic patterns or subtle disease presentations, which are often the most challenging to detect and rightly classify.

The utility of PARS extends beyond lesion detection and segmentation. In clinical settings, rapid and accurate TB estimation could play a pivotal role in treatment planning and monitoring, particularly for checkpoint inhibitor therapies where early response assessment is critical. While PARS currently requires significant expert oversight, future versions could enable semi-automated workflows where the software generates initial segmentations for clinician review, thereby reducing the manual workload without compromising accuracy.

The precision-recall trade-off observed in this study suggests that dynamic thresholding might be implemented to tailor segmentation outputs to specific clinical needs. For instance, lower thresholds could be used for comprehensive disease screening, while higher thresholds might guide more targeted applications such as stereotactic body radiotherapy planning.

This retrospective, single-center study has several limitations. PET/CT scans were acquired on multiple scanners without phantom harmonization was performed. Manual contours from two radiation oncologists served as ground truth, but interobserver agreement was not quantified. The cohort excluded patients with brain-only metastases and came from a single center, which limits generalizability.

In summary, to fully realize its potential, PARS and similar AI tools must address the limitations observed in this study: (1) Enhanced training datasets: Expanding the training set to include diverse lesion types, sizes, and anatomical contexts could improve model generalizability. Annotated data from multi-institutional cohorts, particularly for underrepresented lesion sites, would be invaluable; (2) Expansion of scope: Using information from the available CT scans for lesion contouring might be the next pragmatic step forward; (3) Multi-modal imaging: Integrating data from different imaging modalities, such as MRI or contrast-enhanced CT, could enhance lesion characterization and reduce FPs; (4) Adaptive learning: Models that adapt to patient-specific characteristics, such as lesion growth patterns and metabolic activity changes, could improve segmentation accuracy and TB estimation over time; and (4) Clinical decision support systems: Future iterations of PARS could incorporate predictive analytics, such as estimating the likelihood of treatment response or disease progression based on TB dynamics, to assist medical, radiation and surgical oncologists in treatment planning.

In conclusion, while the PARS model demonstrates promising capabilities for lesion detection and TB estimation, it requires further refinement to overcome site-specific limitations and variability in performance. Incorporating advanced AI techniques, diverse training datasets, and multi-modal imaging could substantially enhance its utility. With continued development, tools like PARS have the potential to change how clinicians monitor and manage metastatic melanoma, ultimately improving patient outcomes.

## Availability of data and material

Collected patient and imaging data are confidential and not available for publication.

## Code availability

Not applicable for this publication.

## Authors' contributions

All authors made significant contributions to this project. The idea and conceptualization of the project were developed by STL, MG and SMC. STL and MG obtained the ethical approval for the project. HSG, MA, SMC, SB, and others supported the manual lesion segmentation. LLF, HSG, and MF applied the PARS model to the imaging data set and conducted all subsequent data analysis and visualization efforts. LLF, HSG and SMC prepared the manuscript. The manuscript was then critically reviewed by all co-authors, incl. CF, PB, and MS. The final version of the manuscript was approved by all authors before submission to PhiRO.

## Funding

Lorenzo Lo Faro received a fellowship grant from the Department of Radiation Oncology, University of Zurich, to enable him to work on this project.

## Declaration of competing interest

The authors declare that they have no known competing financial interests or personal relationships that could have appeared to influence the work reported in this paper.
