# Supplementary material for: Deep learning [18F]-FDG-PET/CT‑based algorithm for tumor burden estimation in metastatic melanoma patients under immunotherapy
Source: Clin Transl Radiat Oncol. 2025 Oct 27;56:101063. doi: 10.1016/j.ctro.2025.101063 (PMC12639252; doi:10.1016/j.ctro.2025.101063)
Supplement: Supplementary Data 1 [file mmc1.docx]

# Supplements

**Table S1.** Individual lesion size estimation agreement between experts and PARS for different anatomical locations.

| **Location** | **Median difference [cc]** | **Median absolute difference [cc]** | **Median relative percentage difference** | **Median absolute relative percentage difference** | **ICC (95% CI)** |
| --- | --- | --- | --- | --- | --- |
| All (n=626) | -0.9 | 1.5 | -34.3% | 44.9% | 0.77 (0.55 – 0.88) |
| Lymph node  (n=213) | -1.3 | 1.9 | -38.8% | 42.5% | 0.74 (0.58 – 0.85) |
| Bone (n=82) | -0.8 | 1.1 | -32.3% | 48.2% | 0.73 (0.53 – 0.84) |
| Liver  (n=95) | -0.2 | 3.9 | -6.1% | 56.4% | 0.90 (0.60 – 0.95) |
| Lung  (n=97) | -0.2 | 0.7 | -22.0% | 38.0% | 0.52 (0.29 – 0.80) |
| Other  (n=139) | -1.4 | 1.6 | -40.4% | 51.3% | 0.58 (0.12 – 0.85) |
